# Increased *IL-10 *mRNA expression in tumor-associated macrophage correlated with late stage of lung cancer

**DOI:** 10.1186/1756-9966-30-62

**Published:** 2011-05-20

**Authors:** Rui Wang, Meng Lu, Jie Zhang, Sufeng Chen, Xiaoyang Luo, Ying Qin, Haiquan Chen

**Affiliations:** 1 Center of Lung Cancer Prevention & Treatment, Department of Thoracic Surgery, Shanghai Cancer Hospital, Fudan University; Department of Oncology, Shanghai Medical College, Fudan University, Shanghai 200032, China; 2 Institute for Nutritional Sciences, Shanghai Institutes for Biological Sciences, Chinese Academy of Sciences; Graduate School of the Chinese Academy of Sciences, 200031, China

**Keywords:** Lung cancer, Tumor associated macrophages, IL-10

## Abstract

**Background:**

Monocyte recruited into the tumor and maturation to tumor-associated macrophage (TAM). *Interleukin-10(IL-10) *is a potent immunosuppressive cytokine, which can be secreted from both primary tumor and stromal cells. However, there are controversies regarding its role in the progression of cancer. So it is important to isolate TAM from tumor cells to study the role of *IL-10 *in the progress of cancer. The aim of our study was to determine whether *IL-10 *expressed by TAM correlated with clinicopathological factors in NSCLC.

**Methods:**

TAM in NSCLC was isolated by short-term culture in serum free medium with the modification to literature reports. The mRNA expression levels of *IL-10*, *cathepsin B*, *cathepsin S*, which were closely related with TAM according to the literatures, were evaluated by Quantitative real-time RT-PCR in 63 NSCLC. The relationships between their expression levels and clinicopathological features were investigated.

**Results:**

We successfully achieved up to 95% purity of TAM, derived from 63 primary lung cancer tissues. TAM expressed high levels of *IL-10*, *cathepsin B *in NSCLC. High levels of *IL-10 *in TAM significantly correlated with stage, tumor size, lymph node metastasis, lymphovascular invasion or histologic poor differentiation.

**Conclusions:**

Our results revealed that TAM with high levels of *IL-10 *expression may play an important role in the progression of non-small cell lung cancer. The data also suggested that TAMs may involve in tumor immunosuppression through overexpressed *IL-10*. Additionally, the phenotype of isolated TAM can be potentially used to predict clinicopathological features as well.

## Background

Tumor-associated macrophages (TAMs) are the most abundant cancer stromal cells involved in the host immune system [1,2]. In recent years, increasing attention has focused on TAMs, unique macrophage populations that play pivotal roles in tumor immunosuppression, and provide a suitable microenvironment for cancer development and progression[3]. TAM infiltration has been found to be correlated with a worse outcome in several malignant tumors [4-9]. The possible mechanism by which TAMs support tumor progression and help the tumor evade immunosurveillance is through the release a spectrum of tumor promoting and immunosuppressive products.

*Interleukin-10(IL-10)*, *cathepsin B *or *cathepsin S *was reported to be closely associated with TAMs in recent literatures [10-12]. *IL-10 *is produced primarily by T cells, B cells, dendritic cells, and monocytes/macrophages[13]. Tumor-associated macrophages form a major component in a tumor, and have been suggested to play an essential role in the complex process of tumor-microenvironment coevolution and tumorigenesis[1]. Previous reports have also shown that TAMs produce high levels of *IL-10*, exhibit little cytotoxicity for tumor cells[14]. However, there are controversies regarding its role in the progression of cancer [15,16]. So it is important to isolate TAM from tumor cells to study the role of *IL-10 *in the progress of cancer.

By using DNA-microarray technology, recent study demonstrated that NSCLC patients with a high expression level of cathepsins in lung cancer tissue (both tumor cells and stroma cells) had a poor outcome [17]. Interestingly, it has been shown that TAM is the primary source of high levels of cathepsin activity in pancreatic, breast and prostate cancer animal models [10-12]. However, the significance of cathepsins expressed by TAM in NSCLC remains unknown.

In the present study, we assessed *IL-10, cathepsin B and cathepsin S *expression in TAMs, freshly isolated from lung tumor tissue, in correlation with clinicopathological factors in NSCLC.

## Materials and methods

### Subject characteristics

63 paired peripheral blood samples and primary lung cancer tissues were collected from patients before or at the time of surgical resection at the Center for Lung Cancer Prevention and Treatment of Shanghai Cancer Hospital from June 2009 to March 2010. Data collected included age, sex, smoking history, histopathological diagnosis, TNM stage, lymphovascular invasion, pleural invasion, and tumor differentiation. Histological diagnoses, presence of lymphovascular invasion(LVI), and grade of differentiation were confirmed by two senior histopathologists. A consent form was signed by every patient or his/her legal representatives. This study was approved by the committees for Ethical Review of Research at Shanghai Cancer Hospital.

Histological diagnosis and grade of differentiation were determined in accordance with the World Health Organization criteria for lung cancer[18]. The pathologic tumor stage (p stage) was determined according to the revised TNM classification of lung cancer[19].

### Isolation of tumor-associated macrophages

TAMs were isolated from solid tumors according to literature reports [20-22]. Briefly, Tumor tissue was cut into 2 mm fragments, followed by collagenase digestion (0.3 mg/ml, Worthington Biochemical Corp, NJ, USA) for 1 h at 37°C. The suspension was filtered through a 70 μm stainless steel wire mesh to generate a single-cell suspension. The suspension was centrifuged and washed twice with PBS. Cells were left to adhere in serum-free RPMI 1640 for 40 min. Nonadherent cells were washed away. Ninety-five percent of the remaining adherent cells were TAMs as assessed by morphology and macrophage specific marker CD68 positivity.

#### Immunofluorescence

TAMs were adhered to 24-well plate , fixed in 4% paraformaldehyde at room temperature for 5 minutes, washed with PBS twice, incubated with 1% BSA at 37°C for 30 minutes to block nonspecific interactions, and then stained with primary antibodies to CD68 (1:100 dilution, sc-20060, Santa Cruz Biotechnology, CA, USA) at 4°C overnight. After several washes with PBS, the cells were incubated in an appropriate, rhodamine-labeled goat anti-mouse secondary antibody(Proteintech Group, Inc, Chicago ,USA) at room temperature for 1 h. Nuclei of all cells were then stained with 4'6-diamidino-2-phenylindole(DAPI). Image was taken at 200 × magnification on an Olympus-IX51 microscope. For each patient, 10 fields were imaged and measured for percentage of macrophage (CD68 positive cells/DAPI stained cells). Immunofluorescence was repeated in three randomly selected patients.

### Preparation normal macrophage

Macrophage (Mφ) was obtained as described previously [20]. In brief, the mononuclear cells were isolated from peripheral blood matched with TAMs by Ficoll-Hypaque density gradient centrifugation (density, 1.077 ± 0.001 g/ml, Axis-Shield, Oslo, Norway) at 450 × g for 30 min at room temperature. The mononuclear cells were washed thrice with PBS and plated at 1 × 10^7 ^in 6-cm tissue culture dishe for 2 h in DMEM alone. Thereafter, the nonadherent cells were washed thrice with warm PBS and the adherent monocytes were cultured in DMEM containing 5% FBS and 25 ng/ml human macrophage colony-stimulating factor((rhM-CSF, PeproTech, Rocky Hill, NJ, USA), The medium was changed every 2 days, and macrophage were obtained after 6 days in vitro cultivation.

### RNA isolation and Quantitative real-time RT-PCR(QRT-PCR)

Total RNA was isolated from TAMs and their matched macrophages by using RNeasy Mini Kit (Qiagen, Valencia, CA, USA) as described by the manufacturer's protocol. For mRNA analysis, an aliquot containing 2 μg of total RNA was transcribed reversely using M-MLV reverse transcriptase (Promega, Madison, WI, USA). Specific primers (Genery, Shanghai, China) were used to amplify cDNA. QRT-PCR was done using SYBR Green PCR master mix (Applied Biosystems, Piscataway, NJ, USA). The primers for QRT-PCR were: *β-actin *forward (F) 5' ACCACA CCTTCTACAATGA3'*, β-actin *reverse(R) 5'GTCATCTTCTCGCGGTTG3'; *IL-10 *F 5' AGAACCT GAAGACCCTCAGGC3', *IL-10 *R 5' CCACGGCCTTGCTCTTGTT 3'; *cathepsin B *F 5' TGCA GCGCTGGGTGGATCTA 3'; *cathepsin B *R 5' ATTGGCCAACACCAGCAGGC 3'; *cathepsin S *F 5' GCTTCTCTTGGT GTCCATAC 3', *cathepsin S *R 5' CATTACTGCGGGAATGAGAC 3'. The amplification protocol consisted of an initial 10 min denaturation step at 95°C, followed by 40 cycles of PCR at 95°C for 15s, 60°C for 1 min and detection by the ABI-Prism 7900HT Sequence Detection System (Applied Biosystems, Foster City, CA, USA). Each sample was assayed in triplicate. The comparative C_T _method (ΔΔC_T _method) was used to determine the quantity of the target sequences in TAM relative both to Mφ (calibrator) and to β-actin (an endogenous control). Relative expression levels were presented as the relative fold change and calculated using the formula: 2 ^-ΔΔCT^= 2-(ΔC_T _^(TAM) ^- ΔC_T _^(^Mφ^) ^where each ΔC_T _=ΔC_T _^target^-ΔC_T_^β-actin^.

### Immunohistochemistry

For exact identification of IL-10 or cathepsin B expression in TAMs, serial sections were used to examine the expression of IL-10, cathepsin B in TAMs. Samples were fixed in 4% formaldehyde in PBS (pH 7.2) and paraffin embedded. 4-μm thickness was cut from each paraffin block. After dewaxing and rehydration, the sections were microwaved for antigen retrieval in 10 mmol/liter citrate buffer (pH 6.0) for 10 min, and then allowed to cool for 1 hour at room temperature. Endogenous peroxidase activity was blocked with hydrogen peroxide; Nonspecific binding was blocked by preincubation with 10% goat serum in PBS for 30 minutes at room temperature. Slides were incubated with the primary antibodies directed against monoclonal anti-human CD68 antibody (1:200 dilution, sc-20060, Santa Cruz, CA, USA), monoclonal anti-human IL-10 antibody (1:100 dilution, BA1201,Boster, WuHan, China) or polyclonal anti-human cathepsin B antibody (1:100 dilution, ab49232, Abcam, MA, USA). The results were visualized using the streptavidine-biotin immunoperoxidase detection kit and AEC chromogen (Maixin Bio, FuZhou, China) based on the manufacturer's instruction. Positive cells stained red. The negative control involved omission of the primary antibody.

### Statistical analysis

Statistical analysis software (Prism 5.0, GraphPad Software Inc, La Jolla, CA, USA and SPSS Version 13.0 software, SPSS Inc, Chicago, IL, USA) was used to perform the analyses. Data are expressed as median (range). The Mann-Whitney test was used for the comparison between TAM and normal macrophage. The correlation between *IL-10 *or *cathepsin B *expression and clinicopathologic factors was analyzed by Mann-Whitney test. Multivariate logistic regression was performed to evaluate the relationships between the pathological stage (with early and late stage as dependent variables) and covariates (age, sex, tobacco use, tumor histology and *IL-10 *expression in TAMs). For this analysis, the median value of *IL-10 *was chosen as the cut-off point for dividing the patients into the two groups. Two-tailed P value less than 0.05 was considered statistically significant.

## Results

### Patients characteristics

The patient characteristics are described in Table 1. Patients (40 males and 23 females) had a mean age of 58.8 ± 1.1 years. Fifty-four patients had a smoking history, and forty-six were non-smokers. Adenocarcinoma was the most common tumor type (54%) followed by squamous cell carcinoma (32%). 30 patients (48%) were stage I (early stage), and the remaining 34 patients were (52%) stages II, III or IV (late stage) of the disease.

**Table 1:** characteristics for the patients included in this study

characteristic		**No. **^**a**^**(N= 63)**	%
Age/years(Median,range)		58 (37-76)	
Sex			
	Male	40	63.5
	Female	23	36.5
Tobacco use			
	Current	22	35
	Former	12	19
	Never	29	46
Histology			
	Adenocarcinoma	34	54
	Squamous cell carcinoma	20	32
	Others^b^	9	14
Stage			
	StageⅠ	30	48
	StageⅡ	11	17
	StageⅢ	17	27
	StageⅣ^c^	5	8
Lymph node metastasis			
	N0	42	67
	N1/N2	21	33
Pleural invasion			
	Negative	43	68
	Positive	20	32
Lymphovascular invasion			
	Negative	51	81
	Positive	12	9
Histologic differentiation			
	Well/Moderate	30	48
	Poor	26	41
	not available^d^	7	11

^a ^Number for all except age.

^b ^Include 2 Large cell carcinoma, 2 Carcinoid, 1 malignant clear cell sugar tumor, 1 Sarcomatoid carcinoma, and 3 malignancy , but type undetermined.

^c^Stage IV was found incidentally during the operation or only for biopsy

^d ^Include Carcinoid, malignant clear cell sugar tumor, Sarcomatoid carcinoma, multiple primary lung cancer.

### Isolation and identification of tumor-associated macrophages

In our study, 71 NSCLC samples were collected and TAMs were successful isolated from all samples. However, cell number of TAMs isolated from 8 NSCLC was inadequate for gene expression analysis, and excluded from this study. So TAMs from 63 NSCLC were finally analyzed. The successful rate was 89%(63/71). Each sample weight ranged from 10 mg to 200 mg and the cell number of TAMs collected ranged from 5 × 10^5 ^to 1 × 10^7 ^per 100 mg tumor tissue.

**Figure 1:**
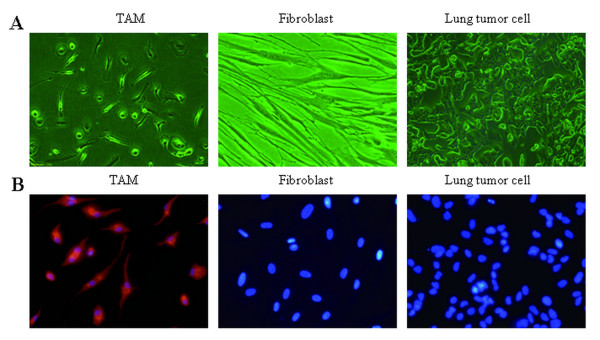
**Characterization of tumor-associated macrophage**. A. Representative cell morphology of tumor-associated macrophages, TAM, fibroblast and lung tumor cell. B. Immunofluorescent was used to distinguish macrophage, fibroblast and lung tumor cell with antibodies targeting *CD68 *(red), nuclei stained with DAPI (blue). Original magnification, × 400.

TAMs from lung cancer tissue had an irregular shape and projections (Figure 1A). To confirm that the cell isolated from the lung cancer tissue were TAMs without contamination by fibroblasts or tumor cells, staining for the macrophage specific marker *CD68 *was performed. Over ninety-five percent of the cells stained positively for each randomly selected patient (Figure 1B).

### The mRNA expression levels of *IL-10, cathepsin B *and *cathepsin S *in normal macrophages

**Figure 2:**
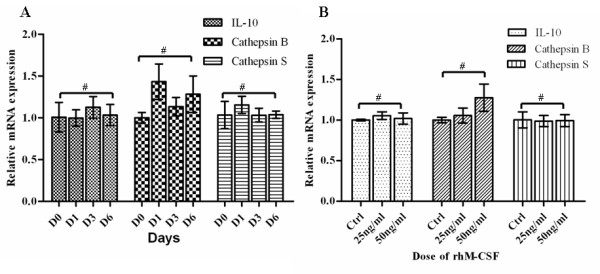
**The mRNA expression levels of IL-10, cathepsin B and cathepsin S in normal macrophages**. Results are given as fold increase in mRNA expression with respect to expression in D0 monocytes. Data were normalized to expression of the β-actin gene. A: Monocytes(D0) was used as a calibrator. B, monocytes culture without rhM-CSF was used as a calibrator (Ctrl). Error bar is SD, Independent experiments were repeated three times, all #p > 0.05(by student t-test).

We performed a time course study to show the expression level of *IL-10*, *cathepsin B *and *cathepsin S *in monocytes changes after culture in medium with rhM-CSF. Our study showed the expression level of IL-10, cathepsin B and cathepsin S showed no significant changes in the time dependent study. (All p > 0.05) (Figure 2A). We also performed dose depedent study of rhM-CSF to see whether the expression level of IL-10, cathepsin B and cathepsin S were affected or not. Our study showed that the dose of rhM-CSF did not affect the expression level of IL-10, cathepsin B and cathepsin S (Figure 2B).

### The mRNA expression levels of *IL-10, cathepsin B *and *cathepsin S *in TAMs

**Figure 3:**
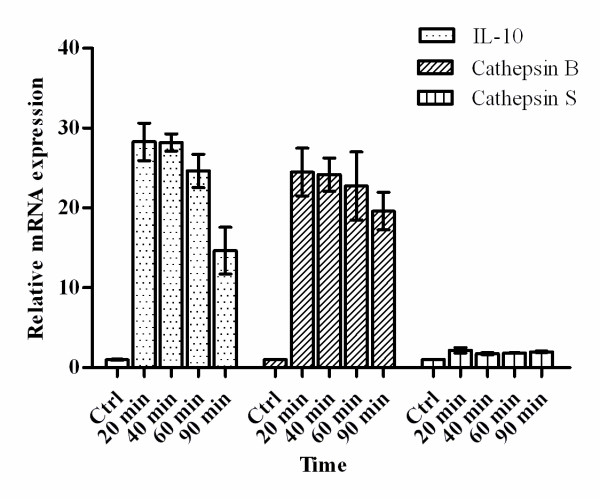
**The mRNA expression levels of *IL-10, cathepsin B *and *cathepsin S *in TAM changes in primary culture**. Results are given as fold increase in mRNA expression with respect to expression in ctrl (normal macrophages). Data were normalized to expression of the *β-actin *gene. Normal macrophages were used as a calibrator. Error bar is SD; Independent experiments were repeated three times.

The mRNA expression levels of *IL-10, cathepsin B *and *cathepsin S *in TAMs were analyzed using QRT-PCR, compared with matched normal macrophages from the 63 patients. To explore the best time point for analyzing the expression level of *IL-10, cathepsin B *and *cathepsin S*, a time course study was done. After adhere to plastic for 20 min, 40 min, 60 min and 90 min, the expression level of IL-10 were: 28.3 ± 2.3; 28.1 ± 1.1; 24.6 ± 2.1; 14.7 ± 2.9 respectively, and the purity of TAMs were: 100%, 97%, 95%, 84% respectively (staining for the macrophage specific marker *CD68 *was performed). After 60 min, tumor cells and fibroblast began to adhere, the purity decreased rapidly. So we chose 40 min as the time point for adherence, which is consistent with previous reports [23] (Figure 3).

**Figure 4:**
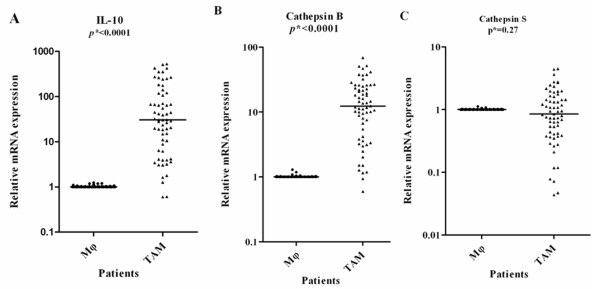
**mRNA from TAMs and matched normal macrophage(Mφ) was analyzed by Quantitative real-time RT-PCR for expression of the indicated genes in 63 NSCLC samples**. Results are given as fold increase in mRNA expression with respect to expression in matched Mφ. Data were normalized to expression of the β-actin gene. Mφ was used as a calibrator. Bars represent median. *p by the Mann-Whitney U test.

Compared with the expression in macrophage, *IL-10 *and *cathepsin B *were significantly upregulated (p < 0.05). After normalized to macrophages, the median values (range) of each gene in TAM were: *IL-10*, 30.5(0.6-530.3) and *cathepsin B*, 11.9(0.6-69.1) (Figure 4 A-B). There were no significant differences in the level of *cathepsin S *between the TAMs(0.85(0.04-4.49))and the macrophages (Figure 4C).

### Immunohistochemistry

**Figure 5:**
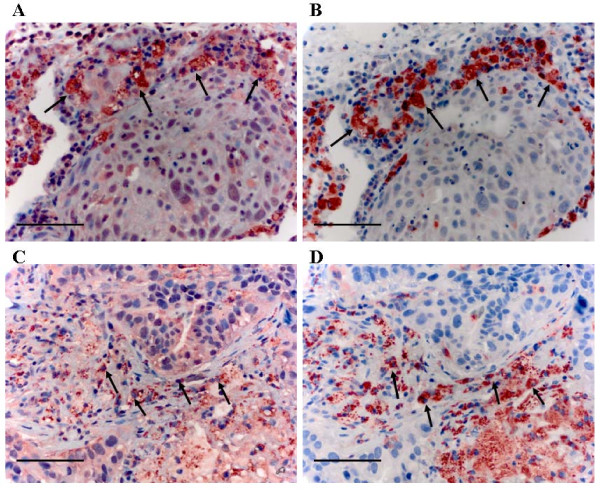
**Immunohistochemical expression of *IL-10*, *cathepsin B *and *CD68 *in macrophage**. A-B, High IL-10 expression in macrophage, A, *IL-10 *staining in macrophage (strong positivity); B, *CD68 *staining. C-D, *Cathepsin B *expression in macrophage; C, *cathepsin B *staining in macrophage (most cells were moderate positivity, only a few cells were strong staining); D, *CD68 *staining. Scale bar indicates 50 μm. Original magnification, × 400.

To confirm whether TAMs express *IL-10 *and *cathepsin B *in protein level, 6 NSCLC (3 late stage (ⅢA) and 3 early stage (Ia- Ib)) were randomly selected to perform IHC using antibody against *CD68*, *IL-10 *and *cathepsin B *on serial sections. We demonstrated that almost all *CD68 *positive cells co-expressing *IL-10*, which in line with the QRT-PCR results that the IL-10 mRNA expression level is high (Figure 5 A-B). The *IL-10 *expression was negative by IHC in 3 early stage NSCLC, which in line with the QRT-PCR results that the *IL-10 *mRNA expression level below the median (30.5) in 3 early stage NSCLC. Expression of cathepsin B in macrophage was observed in 5 of 6 cases. Among macrophages expressing *cathepsin B*, only a small portion of the cells showed strong positive (Figure 5 C-D) and not associated with stage of disease.

### The correlation between *IL-10, cathepsin B *expression in TAM and clinicopathologic factors

The correlation between *IL-10, cathepsin B *expression in TAM and clinicopathologic factors was shown in Table 2. A strongly positive correlation between *IL-10 *mRNA expression in TAM and tumor stage was seen. Increased expression levels of *IL-10 *in TAM were seen in NSCLC patients with late stage (stage II, III and IV). When multivariate logistic regression analysis was performed, *IL-10 *expression in TAMs was shown to be an independent predictive factor for late stage disease (Table 3).

**Table 2:** Genes expression of TAM in relationship with clinicopathological factors

		*IL-10*		*Cathepsin B*	
		
Variables	N	Median(Range)	p* value	Median (Range)	p* value
age					
<58	26	31.3(3.05-530.3)	0.252	10.9(0.9-51.9)	0.41
≥58	37	30.5(0.6-511.6)		14.5(0.6-69.1)	
Gender					
Male	40	31.3(1.3-530.3)	0.607	14.9(0.9-69.1)	0.061
Female	23	19.9(0.6-426.1)		10.1(0.6-37.9)	
Smoking history					
Never	29	30.5(0.6-426.1)	0.699	10.1(0.6-51.9)	0.067
Former or current	34	31.2(1.3-530.3)		14.9(1.5-69.1)	
Histology					
Adenocarcinoma	34	42.9(0.6-530.3)	0.045	12.7(0.6-69.1)	0.41
Squamous cell carcinoma	20	17.1(1.3-354.3)		16.6(1.5-41.7)	
Others	9	41.2(6.4-511.6)		10.2(4.2-26.7)	
Pathological stage					
Stage I	30	9.7(0.6-140.8)	0.016	13.1(0.6-69.1)	0.066
StageⅡ	11	28.9(1.8-511.6)		13.6(3.1-41.7)	
StageⅢ	17	177.7(23.5-530.3)		11.8(1.2-51.9)	
StageⅣ	5	249.9(55.4-429.9)		10.1(3.6-25.9)	
T status					
T1	15	4.1(0.6-263.6)	<0.0001	9.9(0.6-22.7)	0.037
T2-3	48	42.9(1.6-530.3)		14.2(0.9-69.1)	
Lymph node metastasis					
N(+)	21	119.1(6.1-530.3)	<0.0001	13.6(1.2-46.9)	0.466
N(-)	42	19.2(0.6-273.8)		11.1(0.6-69.1)	
Lymphovascular invasion					
LVI(+)	12	93.1(6.2-530.3)	0.01	14.2(0.9-37.8)	0.92
LVI(-)	51	26.5(0.6-429.9)		11.1(0.6-69.1)	
Pleural invasion					
PL(+)	20	55.8(14.9-530.3)	0.002	14.2(0.9-69.1)	0.376
PL(-)	43	19.9(0.6-354.9)		11.1(0.6-51.9)	
Differentiation					
Well or Moderately	30	17.3(0.6-429.9)	0.001	13.0(0.6-69.1)	0.961
poorly	26	113.1(1.6-530.3)		11.9(1.2-37.9)	

*p by the Mann-Whitney U test

**Table 3:** Logistic regression analysis of the association between tumor stage and clinicopathological features (n = 63)

	B	SEM	Chi-squared	p-value	OR (95% CI)
**Sex**	0.241	1.110	0.037	0.847	1.239(0.141-10.922)
**Age**	-0.063	0.040	2.484	0.115	0.939(0.868-1.015)
**Tobacco use**	1.173	1.102	1.133	0.287	3.231(0.373-28.005)
**Histology**	0.292	0.531	0.303	0.582	1.339(0.473-3.793)
**High level *IL-10 *expression in TAM**	2.952	0.742	15.844	0.0001	19.137(4.474-81.859)

The dependent variabl**e is **early- or late-stage group The independent variables included sex (0 = female; 1 = male), age (continuous variable, in yrs), Tobacco use (0 = Current,1 = Former,2 = Never), histology (1 = adenocarcinoma; 2 = squamous cell carcinoma;3 = others) and IL-10 expression (0 = low (<30.5); 1 = high (≥30.5). OR: odds ratio; CI: confidence interval.

The increased mRNA expression of *IL-10 *was also associated with lymph node metastasis, lymphovascular invasion, pleural invasion and poor differentiation (p < 0.0001, p = 0.010, p = 0.017 p = 0.001, respectively).

A correlation between *cathepsin B *mRNA expression in TAM with NSCLC tumor T status was found (p = 0.037). Otherwise, there was no significant relationship between the mRNA expression of *cathepsin B *with any other clinicopathological factors (all p > 0.05).

## Discussion

Increased infiltration of TAMs into NSCLC correlates with a poor prognosis [5,9]. However, the mechanisms for this effect remain unclear. TAM derived molecules that function to suppress immune activation, promote extracellular matrix (ECM) remodeling may play important roles in NSCLC progression.

In the present study, the rational we selected *IL-10, cathepsin B *or *cathepsin S*, is that they were reported to be closely associated with TAMs in recent literatures [10-12,24]. *IL-10 *is widely known as an potent immunosuppressive cytokine associated with cancer [13,25]. It is produced by a number of cells, including tumor cells and TAMs[14,25]. *Cathepsins B, cathepsin S*, proteolytic enzymes, were thought to facilitate the breakdown of basement membranes thereby promoting cancer cell invasion into surrounding normal tissues. TAM expressed *cathepsin B *or *cathepsin S *in pancreatic islet, breast or prostate cancer animal models. In our study, we showed, TAM expressed high levels of *IL-10, cathepsin B*, but not *cathepsin S *in NSCLC.

Our study suggested that increased *IL-10 *expression of TAM in NSCLC patients correlated with late stage disease (stage II, III and IV), lymph node metastases, pleural invasion, lymphovascular invasion and poor differentiation. Although recent animal model studies indicated that *cathepsin B *or *cathepsin S *expressed by TAM play an important role in tumor progression[10,11], and we also found *cathepsin B *upregulated in TAM, we failed to demonstrate any correlation between *cathepsin B *in TAM and stage, lymph nodal metastasis, pleural invasion or differentiation in NSCLC.

TAMs are derived from blood monocytes that are attracted to a tumor by cytokines and chemokines[14]. In the tumor microenvironment, monocytes differentiate into a distinct macrophage phenotype, which is characterized by the production of high level of *IL-10*. TAM with high *IL-10 *expression level may tune inflammatory responses and adaptive Th2 immunity, exhibit anti-inflammatory and tissue remodeling functions and thereby, to favor tumor progression[14]. We demonstrated that NSCLC patients with late stage disease had a higher level of *IL-10 *expression in TAM, which further supports this hypothesis.

*IL-10 *is a potent immunosuppressive factor that may promote lung cancer growth by suppressing macrophage function and enabling tumors to evade immunosurveillance[26]. The potential importance of *IL-10 *in cancer progression is supported by reports of an association between high *IL-10 *levels in serum or in tumors and worse survival in lung cancer patients[15]. However, other authors demonstrated that lack of *IL-10 *expression by the tumor was associated with a worse survival in patients with stage I NSCLC [16]. The reason for these conflicting results might be that, both tumor cells and stromal(including macrophage) cells can secrete *IL-10*. Additionally, Wagner S et al identified that macrophage was the major source of *IL-10 *in gliomas[27]. So it is important to isolate TAM from tumor cells to study the role of *IL-10 *in the progression of cancer. In our study, we demonstrated the phenotype of isolated TAM was closely associated with clinicopathological features. We can predict tumor size, lymph nodal metastasis and pleural invasion through.*IL-10 *expression in isolated TAM. We also found that the high expression of *IL-10 *in TAM was associated with poorly differentiation, which highlighted a significance role of *IL-10 *secreted by TAM in tumor aggressiveness.

A crucial step of cancer invasion and metastasis is the destruction of basement membrane by proteases. Recent studies showed invasion of cancer cell is increased by the proteases secreted from TAMs. *Cathepsin B *or *cathepsin S *has been implicated in the progression of various human cancers, including bladder, breast, prostate and lung cancers [17,28-30]. The cellular source of this protease in human cancers, consisting of both tumor cells and stromal cells (e.g., fibroblasts, endothelial cells, and TAMs), has remained elusive. Studies using animal models have demonstrated that TAMs are the primary source of high levels of *cathepsin B *or *cathepsin S *in prostate, pancreatic islet cancers, and mammary tumors, and its expression by TAMs plays critical roles in multiple stages of tumor growth and metastasis[10,12,29]. Our studies demonstrated that TAM isolated from NSCLC overexpressed *cathepsin B *but not *cathepsin S*, and the *cathepsin B *levels were not associated with NSCLC stage, lymph metastasis, lymphovascular invasion or histological differentiation.

## Conclusions

*Interleukin-10 *expression in tumor-associated macrophages correlates with disease aggressiveness of non-small cell lung cancer. We plan to conduct further studies to analyze the relationship between *IL-10 *in TAM and survival. The study concerning regulation of *IL-10 *in TAM is ongoing too. It will help to clarify and understand the possible mechanisms *IL-10 *secreted by TAM in the progression of NSCLC.

## Authors' contributions

RW and ML designed and performed the experiment and prepared the manuscript. HQC and JZ supervised the project. YQ, SFC, XYL acquired their authorship for assistance in collecting samples and analyzing data. All authors have read and approved the final manuscript.

## Competing interests

The authors declare that they have no competing interests.

## References

[B1] Pollard JW Tumour-educated macrophages promote tumour progression and metastasis Nat Rev Cancer 2004 4 1 71 78 10.1038/nrc1256 14708027

[B2] Balkwill F Mantovani A Inflammation and cancer: back to Virchow? Lancet 2001 357 9255 539 545 10.1016/S0140-6736(00)04046-0 11229684

[B3] Joyce JA Pollard JW Microenvironmental regulation of metastasis Nat Rev Cancer 2009 9 4 239 252 10.1038/nrc2618 19279573 PMC3251309

[B4] Ohno S Ohno Y Suzuki N Kamei T Koike K Inagawa H Kohchi C Soma G Inoue M Correlation of histological localization of tumor-associated macrophages with clinicopathological features in endometrial cancer Anticancer Res 2004 24 5C 3335 3342 15515429

[B5] Takanami I Takeuchi K Kodaira S Tumor-associated macrophage infiltration in pulmonary adenocarcinoma: association with angiogenesis and poor prognosis Oncology 1999 57 2 138 142 10.1159/000012021 10461061

[B6] Leek RD Lewis CE Whitehouse R Greenall M Clarke J Harris AL Association of macrophage infiltration with angiogenesis and prognosis in invasive breast carcinoma Cancer Res 1996 56 20 4625 4629 8840975

[B7] Lissbrant IF Stattin P Wikstrom P Damber JE Egevad L Bergh A Tumor associated macrophages in human prostate cancer: relation to clinicopathological variables and survival Int J Oncol 2000 17 3 445 451 10938382 10.3892/ijo.17.3.445

[B8] Hanada T Nakagawa M Emoto A Nomura T Nasu N Nomura Y Prognostic value of tumor-associated macrophage count in human bladder cancer Int J Urol 2000 7 7 263 269 10.1046/j.1442-2042.2000.00190.x 10910229

[B9] Chen JJ Lin YC Yao PL Yuan A Chen HY Shun CT Tsai MF Chen CH Yang PC Tumor-associated macrophages: the double-edged sword in cancer progression J Clin Oncol 2005 23 5 953 964 15598976 10.1200/JCO.2005.12.172

[B10] Gocheva V Wang HW Gadea BB Shree T Hunter KE Garfall AL Berman T Joyce JA IL-4 induces cathepsin protease activity in tumor-associated macrophages to promote cancer growth and invasion Genes Dev 2010 24 3 241 255 10.1101/gad.1874010 20080943 PMC2811826

[B11] Lindahl C Simonsson M Bergh A Thysell E Antti H Sund M Wikstrom P Increased levels of macrophage-secreted cathepsin S during prostate cancer progression in TRAMP mice and patients Cancer Genomics Proteomics 2009 6 3 149 159 19487544

[B12] Vasiljeva O Papazoglou A Kruger A Brodoefel H Korovin M Deussing J Augustin N Nielsen BS Almholt K Bogyo M Tumor cell-derived and macrophage-derived cathepsin B promotes progression and lung metastasis of mammary cancer Cancer Res 2006 66 10 5242 5250 10.1158/0008-5472.CAN-05-4463 16707449

[B13] de Waal Malefyt R Yssel H Roncarolo MG Spits H de Vries JE Interleukin-10 Curr Opin Immunol 1992 4 3 314 320 10.1016/0952-7915(92)90082-P 1418711

[B14] Coffelt SB Hughes R Lewis CE Tumor-associated macrophages: effectors of angiogenesis and tumor progression Biochim Biophys Acta 2009 1796 1 11 18 19269310 10.1016/j.bbcan.2009.02.004

[B15] Hatanaka H Abe Y Kamiya T Morino F Nagata J Tokunaga T Oshika Y Suemizu H Kijima H Tsuchida T Clinical implications of interleukin (IL)-10 induced by non-small-cell lung cancer Ann Oncol 2000 11 7 815 819 10.1023/A:1008375208574 10997808

[B16] Soria JC Moon C Kemp BL Liu DD Feng L Tang X Chang YS Mao L Khuri FR Lack of interleukin-10 expression could predict poor outcome in patients with stage I non-small cell lung cancer Clin Cancer Res 2003 9 5 1785 1791 12738735

[B17] Cordes C Bartling B Simm A Afar D Lautenschlager C Hansen G Silber RE Burdach S Hofmann HS Simultaneous expression of Cathepsins B and K in pulmonary adenocarcinomas and squamous cell carcinomas predicts poor recurrence-free and overall survival Lung Cancer 2009 64 1 79 85 10.1016/j.lungcan.2008.07.005 18760860

[B18] Beasley MB Brambilla E Travis WD The 2004 World Health Organization classification of lung tumors Semin Roentgenol 2005 40 2 90 97 10.1053/j.ro.2005.01.001 15898407

[B19] Detterbeck FC Boffa DJ Tanoue LT The new lung cancer staging system Chest 2009 136 1 260 271 10.1378/chest.08-0978 19584208

[B20] Solinas G Schiarea S Liguori M Fabbri M Pesce S Zammataro L Pasqualini F Nebuloni M Chiabrando C Mantovani A Tumor-conditioned macrophages secrete migration-stimulating factor: a new marker for M2-polarization, influencing tumor cell motility J Immunol 2010 185 1 642 652 10.4049/jimmunol.1000413 20530259

[B21] Sierra JR Corso S Caione L Cepero V Conrotto P Cignetti A Piacibello W Kumanogoh A Kikutani H Comoglio PM Tumor angiogenesis and progression are enhanced by Sema4D produced by tumor-associated macrophages J Exp Med 2008 205 7 1673 1685 10.1084/jem.20072602 18559453 PMC2442644

[B22] Duff MD Mestre J Maddali S Yan ZP Stapleton P Daly JM Analysis of gene expression in the tumor-associated macrophage J Surg Res 2007 142 1 119 128 10.1016/j.jss.2006.12.542 17597158

[B23] Biswas SK Gangi L Paul S Schioppa T Saccani A Sironi M Bottazzi B Doni A Vincenzo B Pasqualini F A distinct and unique transcriptional program expressed by tumor-associated macrophages (defective NF-kappaB and enhanced IRF-3/STAT1 activation) Blood 2006 107 5 2112 2122 10.1182/blood-2005-01-0428 16269622

[B24] Mohamed MM Cavallo-Medved D Rudy D Anbalagan A Moin K Sloane BF Interleukin-6 increases expression and secretion of cathepsin B by breast tumor-associated monocytes Cell Physiol Biochem 2010 25 2-3 315 324 10.1159/000276564 20110692 PMC3030463

[B25] Salazar-Onfray F Interleukin-10: a cytokine used by tumors to escape immunosurveillance Med Oncol 1999 16 2 86 94 10.1007/BF02785841 10456656

[B26] Torisu-Itakura H Lee JH Huynh Y Ye X Essner R Morton DL Monocyte-derived IL-10 expression predicts prognosis of stage IV melanoma patients J Immunother 2007 30 8 831 838 10.1097/CJI.0b013e318158795b 18049335

[B27] Wagner S Czub S Greif M Vince GH Suss N Kerkau S Rieckmann P Roggendorf W Roosen K Tonn JC Microglial/macrophage expression of interleukin 10 in human glioblastomas Int J Cancer 1999 82 1 12 16 10.1002/(SICI)1097-0215(19990702)82:1<12::AID-IJC3>3.0.CO;2-O 10360813

[B28] Eijan AM Sandes EO Riveros MD Thompson S Pasik L Mallagrino H Celeste F Casabe AR High expression of cathepsin B in transitional bladder carcinoma correlates with tumor invasion Cancer 2003 98 2 262 268 10.1002/cncr.11493 12872343

[B29] Fernandez PL Farre X Nadal A Fernandez E Peiro N Sloane BF Shi GP Chapman HA Campo E Cardesa A Expression of cathepsins B and S in the progression of prostate carcinoma Int J Cancer 2001 95 1 51 55 10.1002/1097-0215(20010120)95:1<51::AID-IJC1009>3.0.CO;2-J 11241311

[B30] Maguire TM Shering SG Duggan CM McDermott EW O'Higgins NJ Duffy MJ High levels of cathepsin B predict poor outcome in patients with breast cancer Int J Biol Markers 1998 13 3 139 144 10079387 10.1177/172460089801300303

